# Evidence for the Cytoplasmic Localization of the L-α-Glycerophosphate Oxidase in Members of the “*Mycoplasma mycoides* Cluster”

**DOI:** 10.3389/fmicb.2019.01344

**Published:** 2019-06-19

**Authors:** Melanie Schumacher, Pamela Nicholson, Michael H. Stoffel, Suchismita Chandran, Adonis D’Mello, Li Ma, Sanjay Vashee, Joerg Jores, Fabien Labroussaa

**Affiliations:** ^1^Institute of Veterinary Bacteriology, University of Bern, Bern, Switzerland; ^2^Division of Veterinary Anatomy, University of Bern, Bern, Switzerland; ^3^J. Craig Venter Institute, Rockville, MD, United States; ^4^Institute for Genome Sciences, University of Maryland School of Medicine, Baltimore, MD, United States

**Keywords:** “*Mycoplasma mycoides* cluster, ” synthetic genomics, mycoplasma virulence traits, L-α-glycerophosphate oxidase, Triton X-114, scanning electron microscopy

## Abstract

Members of the “*Mycoplasma mycoides* cluster” are important animal pathogens causing diseases including contagious bovine pleuropneumonia and contagious caprine pleuropneumonia, which are of utmost importance in Africa or Asia. Even if all existing vaccines have shortcomings, vaccination of herds is still considered the best way to fight mycoplasma diseases, especially with the recent and dramatic increase of antimicrobial resistance observed in many mycoplasma species. A new generation of vaccines will benefit from a better understanding of the pathogenesis of mycoplasmas, which is very patchy up to now. In particular, surface-exposed virulence traits are likely to induce a protective immune response when formulated in a vaccine. The candidate virulence factor L-α-glycerophosphate oxidase (GlpO), shared by many mycoplasmas including *Mycoplasma pneumoniae*, was suggested to be a surface-exposed enzyme in *Mycoplasma mycoides* subsp. *mycoides* responsible for the production of hydrogen peroxide directly into the host cells. We produced a *glpO* isogenic mutant GM12::YCpMmyc1.1-*ΔglpO* using in-yeast synthetic genomics tools including the tandem-repeat endonuclease cleavage (TREC) technique followed by the back-transplantation of the engineered genome into a mycoplasma recipient cell. GlpO localization in the mutant and its parental strain was assessed using scanning electron microscopy (SEM). We obtained conflicting results and this led us to re-evaluate the localization of GlpO using a combination of *in silico* and *in vitro* techniques, such as Triton X-114 fractionation or tryptic shaving followed by immunoblotting. Our *in vitro* results unambiguously support the finding that GlpO is a cytoplasmic protein throughout the “*Mycoplasma mycoides* cluster.” Thus, the use of GlpO as a candidate vaccine antigen is unlikely to induce a protective immune response.

## Introduction

The “*Mycoplasma mycoides* cluster” encompasses five pathogenic members which cause livestock diseases including contagious caprine and bovine pleuropneumonia, mastitis, septicemia, arthritis, and pneumonia in ruminants (Fischer et al., 2012). With the recent and dramatic increase of antimicrobial resistance observed in many *Mycoplasma* species (Gautier-Bouchardon, 2018), vaccination of animals is considered the most cost-effective method to eradicate mycoplasma diseases (Nicholas et al., 2009). However, most of the commercial mycoplasma vaccines were developed using empirical approaches and are often reported to have limited efficacy, duration of immunity and side effects (Nicholas et al., 2009). The emergence of synthetic genomics techniques (Lartigue et al., 2009; Chandran et al., 2014; Tsarmpopoulos et al., 2016) has provided a complete and functional genetic platform for the study of mycoplasmas (Lartigue et al., 2014; Labroussaa et al., 2016). In addition to the development of tailored and effective vaccines, these tools allow for the production of isogenic mutants providing a unique opportunity to improve the understanding of host–pathogen interactions and physiological pathways in *Mycoplasmas* (Schieck et al., 2016).

In the absence of a cell wall, proteins such as lipoproteins present at the mycoplasma cell surface are likely to directly interact with host cells during infections (Rottem, 2003). Currently, little is known about the factors and mechanisms driving *Mycoplasma* pathogenicity. The only virulence trait in *Mycoplasma mycoides* that has been confirmed *in vivo* involves the capsular polysaccharide (Jores et al., 2018), which was shown *in vitro* to play a role in membrane integrity and adhesion to host cells (Schieck et al., 2016). More recently, the activity of the *Mycoplasma* Ig binding-*Mycoplasma* Ig protease (MIB-MIP) system (Arfi et al., 2016) in host–pathogen interactions has also been observed in infected animals with severe septicemia (Jores et al., 2018). Additionally, the characterization of mycoplasma “surfaceomes” allowed for the identification of many putative surface-associated or surface-exposed proteins (Krasteva et al., 2014; Reolon et al., 2014). Among them, several mycoplasma lipoproteins have been proposed to play a role in mycoplasma pathogenicity, i.e., adhesion to host cells or immune evasion (Chambaud et al., 1999; Citti et al., 2010; Browning et al., 2011; Christodoulides et al., 2018). Many transporters were also identified at the cell surface and are seen as an adaptive trait of mycoplasmas associated with their parasitic lifestyle (Razin et al., 1998). Indeed, the regressive and convergent evolution that shaped and minimized their genomes, as previously shown for the “*Mycoplasma mycoides* cluster” (Lo et al., 2018), forced mycoplasmas to scavenge for high-energy compounds and metabolites during host infection. Among these, glycerol and glycerol-related products are imported using active transporters, namely the glycerol facilitator factor GlpF (Pilo et al., 2005; Großhennig et al., 2013) or the ATP-binding cassette (ABC) transporter of the Gts family (Vilei and Frey, 2001). The latter one is composed of four genes, namely *gtsA*, *gtsB*, *gtsC*, and *gtsD* (*gtsABCD* cluster), and was described to encode a highly efficient transporter in highly virulent strains of *Mycoplasma mycoides* subsp. *mycoides* (Vilei and Frey, 2001). Interestingly, mycoplasmas were suggested to be able to hijack this glycerol uptake pathway to enhance their virulence (Blötz and Stülke, 2017). The production of hydrogen peroxide (H_2_O_2_) as a by-product of the glycerol assimilation was associated *in vitro* with the induction of cytotoxic effects on epithelial cells (Pilo et al., 2005; Hames et al., 2009). A glycerol kinase (GlpK) was shown to phosphorylate the imported glycerol into glycerol-3-phosphate, which in turn is oxidized into dihydroxyacetone phosphate (DHAP) and H_2_O_2_. The enzyme responsible for the release of H_2_O_2_ was identified as the L-α-glycerophosphate oxidase (GlpO). It appears to be present in many mycoplasma species and was always found associated in a so-called *glpFKO* cluster in members of the “*Mycoplasma mycoides* cluster.” GlpO was suggested to be surface-exposed in *Mmm* in order to facilitate the production of H_2_O_2_ in the close vicinity of the host cells during *Mmm* infections while limiting toxic effects on the mycoplasma cells (Pilo et al., 2005). Therefore, GlpO has long been considered as an antigen of choice to potentially elicit protective immunity. However, GlpO has also been characterized as a cytosolic dimeric enzyme in other bacteria (Claiborne, 1986; Parsonage et al., 1998) and notably, it was reported to be predominately located in the cytoplasm of *Mycoplasma pneumoniae* (Hames et al., 2009). Such inconsistencies question the localization of GlpO in representative members of the whole “*Mycoplasma mycoides* cluster.”

In this study, an isogenic *Mycoplasma mycoides* subsp. *capri* GM12 mutant, deficient for *glpO*, was generated and analyzed by scanning electron microscopy (SEM) using its parental strain as a control. Hydrogen peroxide production was also quantified for both strains *in vitro* and compared to other mycoplasma species including several strains of *Mycoplasma feriruminatoris*. Lastly, phase partitioning followed by immunoblotting was performed for all members of the “*Mycoplasma mycoides* cluster” and the results were compared to *in silico* analyses of GlpO amino acids (aa) sequences. Collectively, our results clearly demonstrate that GlpO is a cytoplasmic enzyme across the members of the “*Mycoplasma mycoides* cluster.”

## Materials and Methods

### *Mycoplasma* and Yeast Strains

The mycoplasma strains used in this study are listed in the Table 1. All strains were cultured in SP5 medium at 37°C (Labroussaa et al., 2016). Tetracycline (5 μg/ml) was added to the medium when needed.

**TABLE 1:** *Mycoplasma* species used in this study.

*Mycoplasma* species	Strain	Origin	Isolated	Host	Accession number	References
*M. leachii*	99/014/06	Australia	1999	Calf	FR668087.1	Djordjevic et al., 2001
*M. leachii*	PG50^T^	Australia	1963	Cattle	CP002108.1	Wise et al., 2012
*M. mycoides* subsp. *capri*	D2083/91	Switzerland	1991	Goat		Thiaucourt et al., 2000
*M. mycoides* subsp. *capri*	95010	France	1995	Goat	FQ377874.1	Thiaucourt et al., 2011
*M. mycoides* subsp. *capri*	D2503/91	Switzerland	1991	Goat		Vilei et al., 2006
*M. mycoides* subsp. *capri*	D2082/91; D2482/91	Switzerland	1991	Goat		Vilei et al., 2006
*M. mycoides* subsp. *capri*	GM12	USA	1979	Goat	CP001621.1	Lartigue et al., 2009
*M. capricolum* subsp. *capricolum*	6443.90	France	1990	Goat		Vilei et al.,2006
*M. capricolum* subsp. *capricolum*	4146	France	1980	Goat		Fischer et al.,2012
*M. capricolum* subsp. *capricolum*	California kid^T^; ATCC 27343	USA	1955	Goat	CP000123.1	Cordy et al., 1955
*M. feriruminatoris*	G5847^T^	Berlin	1993	Alpine Ibex		Jores et al.,2013
*M. feriruminatoris*	8756-C13	USA	<1987	Goat		Manso-Silván et al., 2007
*M. feriruminatoris*	G5813/1+2; 322/93	Berlin	1993	Alpine Ibex		Fischer et al., 2012
*M. feriruminatoris*	G1650; 27/94	Berlin	1993	Alpine Ibex		Schnee et al., 2011
*M. feriruminatoris*	G1705; 28/94	Berlin	1993	Alpine Ibex		Fischer et al., 2012
*M. feriruminatoris*	14/OD_0492	Switzerland	2014	Alpine Ibex		This study
*M. feriruminatoris*	14/OD_0535	Switzerland	2014	Alpine Ibex		This study
*M. capricolum* subsp. *capripneumoniae*	F38^T^	Kenya	1976	Goat	LN515398.1	Falquet et al., 2014
*M. mycoides* subsp. *mycoides*	Afadé	Cameroon	1968	Cattle	LAEX01	Fischer et al., 2015
*M. mycoides* subsp. *mycoides*	Gladysdale	Australia	<1964	Cattle	CP002107	Wise et al.,2012
*M. mycoides* subsp. *mycoides*	B237	Kenya	1987	Cattle	LAEW01	Fischer et al., 2015

*Saccharomyces cerevisiae* strain VL6-48N (*MATα trp1-Δ1 ura3-Δ1 ade2-101 his3-Δ200 lys2 met14 cir°*) (Larionov et al., 1997) containing the 1.08 Mb genome of *Mycoplasma mycoides* subsp. *capri* (*Mmc*) (Lartigue et al., 2009) was used in this study. Construction of the isogenic mutant GM12::YCpMmyc1.1-*ΔglpO* was performed using the tandem repeat coupled with endonuclease cleavage (TREC) approach as previously described (Noskov et al., 2010). Yeast cells were cultured at 30°C in either standard rich medium (YPDA, Clontech) or minimal synthetic medium (SD, Clontech).

### Generation of a *Mycoplasma mycoides* subsp. *capri* Strain GM12::YCpMmyc1.1-*ΔglpO*

Construction of the isogenic mutant GM12::YCpMmyc1.1-*ΔglpO* was accomplished using the TREC approach as previously described (Noskov et al., 2010). Specifically, the gene *glpO* at position 286,008–287,173 in the parental genome of GM12::YCpMmyc1.1 was deleted. This deletion was confirmed by PCR and Sanger sequencing of the PCR product. Total DNA was extracted, and genome integrity was verified by multiplex PCR and pulsed-field gel electrophoresis (PFGE) as previously described (Labroussaa et al., 2016). The genome GM12::YCpMmyc1.1-*ΔglpO* was isolated in agarose plugs and transplanted back into the restriction free *M. capricolum* subsp. *capricolum* (McapΔRE) recipient cells as described before (Labroussaa et al., 2016). Resulting transplants were selected on SP4 (Tully et al., 1977) agar medium supplemented with 5 μg/mL tetracycline. The expected genotype was verified again using multiplex PCR and PFGE as outlined above. The GM12::YCpMmyc1.1-*ΔglpO* genome was sequence-confirmed using Illumina next generation sequencing.

### Expression and Purification of Recombinant GlpO

Synthetic genes encoding full-length GlpO_GM12_, N-terminal GlpO_GM12_, and C-terminal GlpO_GM12_ were codon-optimized for *Escherichia coli* expression and inserted into the expression vector pET28a(+) (Novagen) by GenScript. The full-length gene encoded all 387 aa (*glpO*_GM12_), while the N-terminal GlpO included the first 193 aa (*NT-glpO*_GM12_) and the C-terminal GlpO the remaining 194 aa (*CT-glpO*_GM12_). The plasmids containing *glpO*_GM12_, *NT-glpO*_GM12_ or *CT-glpO*_GM12_ were transformed into *E. coli* BL21(DE3). Recombinant clones were grown in Lysogeny Broth (LB) containing 40 μg/mL kanamycin at 37°C with 220 rpm until an optical density (OD, measured at 600 nm) of 0.6 was reached. Expression of the recombinant His_6_-tagged GlpO proteins was induced by the addition of isopropyl-β-D-thiogalactopyranoside (IPTG) to the medium at a final concentration of 1 mM and continued shaking at 37°C for 4 h. The culture was centrifuged at 4,000 × *g* for 20 min at room temperature (RT) and the cell pellets were frozen at -20°C. Purification of the His_6_-tagged GlpO proteins were performed using Ni^2+^-nitrilotriacetic acid (Ni-NTA) metal-affinity chromatography matrices (Qiagen) under denaturing conditions and according to the manufacturer’s guidelines. The eluted recombinant proteins were then loaded onto ultrafiltration centrifugal devices containing a polyethersulfone membrane (Thermo Fisher Scientific) for buffer exchange to phosphate buffered saline (PBS).

### Quantification of H_2_O_2_ Production

Hydrogen peroxide production was determined as previously published (Tsarmpopoulos et al., 2016) using the MQuant peroxide test (Merck) which has a detection range of 0.5–25 μg of H_2_O_2_ per mL of solution. Mycoplasma strains were grown in 5 mL cultures. The supernatant was discarded, and the cells were washed twice with incubation buffer (67.6 mM HEPES, 140 mM NaCl, 7 mM MgCl_2_, pH 7.3). The cells were re-suspended in 1.5 mL incubation buffer, transferred into a fresh microcentrifuge tube and centrifuged at 8,600 × *g* at 4°C for 10 min. The pellet was washed with 1 mL incubation buffer and finally resuspended in 4 mL of the fresh incubation buffer. Aliquots of 1 mL were adjusted to an OD_550_ = 1.0. The cell suspensions were incubated at 37°C for 1 h and then glycerol was added to a final concentration of 100 μM. Corresponding aliquots without any added carbon source served as controls. The test strips were dipped into the suspensions for 1 s at the indicated time points and the results were recorded.

### Scanning Electron Microscopy (SEM)

The experiment was performed as previously described (Pilo et al., 2005) with only a few modifications. Briefly, cells of the GM12::YCpMmyc1.1-*ΔglpO* mutant strain and its parental strain, grown in SP5 medium to stationary phase, were centrifuged at 4,000 × *g* for 15 min at 4°C, washed twice with PBS before being re-suspended in 1 mL PBS. The samples were mixed with 16% methanol-free formaldehyde (Thermo Fisher Scientific) to a final concentration of 4% (v/v). Forty microliters of fixed culture was centrifuged on to gold-sputtered and poly-L-Lysine-coated coverslips using a cytospin centrifuge (Hettich Universal 320, Tuttlingen, Germany) at 125 × *g* for 5 min at RT. Thereafter, 0.1% BSA-c/PBS (Aurion, ANAWA Trading, Wangen, Switzerland) and 0.05 M glycine were used to block non-specific binding sites and the free aldehydes. The coverslips were mounted on metal stubs with conductive adhesive tabs (Ted Pella, Redding, CA, United States) and sputter-coated by electron beam evaporation with approximately 8 nm of platinum/carbon (MED020, BalTec, Balzers, Liechtenstein). Two-step immuno-labeling was carried out using a rabbit polyclonal anti-GlpO antibody followed by a 15 nm colloidal gold-conjugated goat anti-rabbit IgG (British Biocell International, United Kingdom) as previously described (Pilo et al., 2005). Control experiments included omission of the primary antibody incubation and use of an irrelevant rabbit primary antibody targeting the alpha smooth muscle actin (Abcam).

### Triton X-114 Partitioning

The Triton X-114 phase partitioning experiments were developed based on previously published protocols (Bordier, 1981; Regula et al., 2000; Krasteva et al., 2014). The *Mycoplasma* cells were recovered by centrifugation at 10,000 × *g* for 10 min at 4°C and were subsequently washed twice with TE buffer (10 mM Tris, 1 mM EDTA, pH 8.0). The pellet was resuspended in 1,800 μL of cold re-suspension buffer (154 mM NaCl, 10 mM Tris, pH 7.4) supplemented with EDTA-free protease inhibitor (Merck) followed by the addition of 200 μL ice-cold 10% Triton X-114 (Sigma-Aldrich). The samples were thoroughly dissolved by vortexing for 30 min followed by rotation for 60 min at 4°C. Prior to phase separation, 100 μL of each sample was retained to serve as the total protein content of the sample. The samples were centrifuged at 12,000 × *g* for 5 min at 4°C and the clear supernatant was transferred to a fresh cold tube followed by 5 min incubation at 37°C. Visible phase separation (a clear viscous lower detergent (TX) phase sharply delineated from an upper aqueous (AQ) phase) was achieved by centrifugation at 8,000 × *g* for 3 min at RT. The upper AQ phase was transferred into a new tube and washed using 1/10th the volume of cold 10% Triton X-114, while cold resuspension buffer was added to a final volume of 2 mL to condense the lower TX-phase. The samples were incubated on ice for 5 min, followed by 5 min incubation at 37°C. Phase separation was again performed by centrifugation at 8,000 × *g* for 3 min at RT. The phase separation was repeated two times and then the AQ-phase was then stored at -20°C until further use. Meanwhile, 1,800 μL cold 100% methanol was added to the TX-Phase, mixed and stored at -20°C. After at least 24 h, they were centrifuged at 12,000 × g for 10 min at 4°C. The pellets were resuspended in 230 μL of 2 × SDS-Sample Buffer. Experiments that required culturing of the stated *Mmm* and *M. capricolum* subsp. *capripneumoniae* (*Mccp*) strains were conducted in a biosafety level 3 laboratory.

### Immunoblotting

Immunoblotting was performed as previously described (Jores et al., 2019). Briefly, protein samples were separated by sodium dodecyl sulfate–polyacrylamide gel electrophoresis (SDS-PAGE) using 12% polyacrylamide gels. The separated proteins were either directly subjected to Coomassie brilliant blue staining or transferred to nitrocellulose membranes using the Trans-Blot SD semi-dry transfer system (Bio-rad) at 18 V in Towbin transfer buffer (25 mM Tris, 192 mM Glycine and 20% (v/v) methanol). After membrane blocking using 5% milk powder or 2% bovine serum albumin (BSA), the membranes were probed with either rabbit anti-GlpO serum (Pilo et al., 2005) diluted at 1:5,000, rabbit anti-LppQ primary antibody (Bonvin-Klotz et al., 2008) diluted at 1:2,000 or rabbit anti-beta Galactosidase polyclonal antibody diluted at 1:5,000 followed by incubation with a horseradish peroxidase (HRP) labeled donkey anti-rabbit IgG (H+L) (Thermo Fisher Scientific) diluted at 1:10,000. Probed protein bands were detected using chemiluminescence (Thermo Fisher Scientific) and were documented using a Vilber-FusionFX-Machine (Vilber Lourmat SAS, France).

### *In silico* Analyses

**FIGURE 1:**
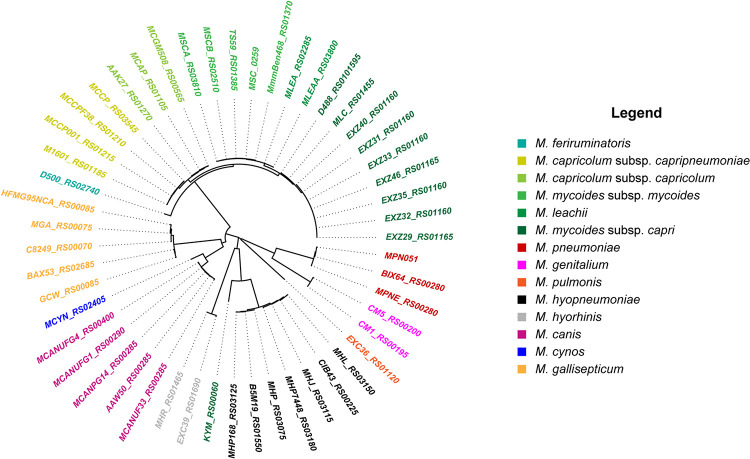
Neighbor-Joining phylogenetic tree of GlpO sequences from 12 *Mycoplasma* species. Identifiers used are locus tags from respective genomes. Coloring and labeling was performed in R using the ape package (Paradis and Schliep, 2019).

Molligen^1^ (Barre et al., 2004) and MBGD^2^ (Uchiyama et al., 2015) databases were used for the comparative genomics analysis looking at the presence and organization of the *glpFKO* and *gtsABCD* clusters. All GlpO sequences were identified from NCBI database using the top BLAST hit per genome, using an E-value cut-off of 1e-5. They were all retrieved and aligned using the ClustalW algorithm in MEGA7 (Kumar et al., 2016). The resulting phylogenetic tree was constructed using the Neighbor-Joining method. In order to improve the overall clarity of the Figure 1, only GlpO sequences that were not 100% identical (at the amino acids level) were used to construct the phylogenetic tree. Sequence information is provided in Supplementary Table S1.

The search for transmembrane domains in GlpO sequences was performed using TMHMM2^3^ (Krogh et al., 2001) and TMpred^4^ (Hofmann and Stoffel, 1993). The search for signal peptides was done using SignalP 5.0^5^ (Almagro Armenteros et al., 2019). In addition, we used PSORTb v3.0^6^ (Yu et al., 2010) for the prediction of subcellular localization of GlpO sequences as it enables specific prediction in *Mycoplasma* species. All GlpO sequences were processed on PSORTb’s online server using the advanced gram stain option of negative without outer membrane, using the long output format. Complete PSORTb data are also available in the Supplementary Table S1.

## Results

### The GlpO Gene Is Present in All Species of the *Mycoplasma mycoides* Cluster

**FIGURE 2:**
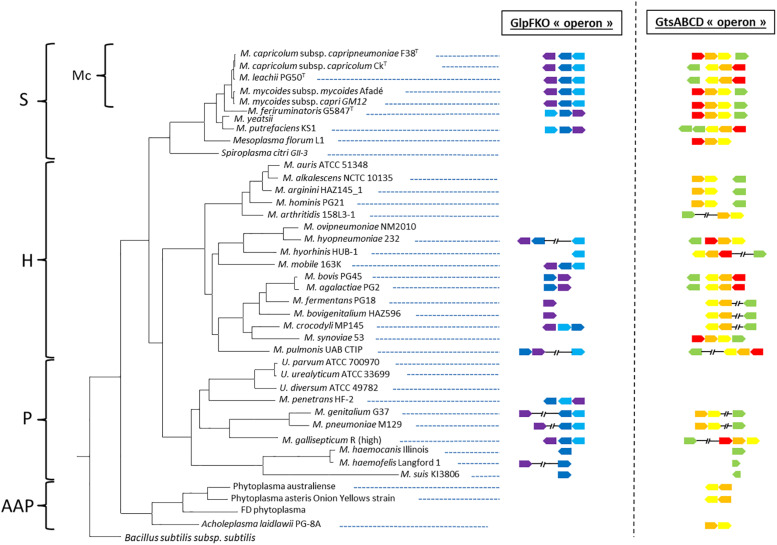
Distribution of the *glpFKO* and *gtsABCD* clusters for different species spanning the entire class of *Mollicutes*. The presence of a gene is indicated by an arrow mimicking the coding sequence orientation and colored in violet (*glpF*), dark blue (*glpK*), light blue (*glpO*), red (*gtsA*), orange (*gtsB*), yellow (*gtsC*), and green (*gtsD*). Main phylogenetic groups are indicated: S, Spiroplasma; Mc, *Mycoides* cluster; H, Hominis; P, Pneumoniae; and AAP, Acholeplasma/Phytoplasma.

We first retrieved 200 GlpO amino acids sequences from NCBI (May 2019). To do so, we acquired 207 mycoplasma genomes consisting of 14 *M. mycoides subsp. mycoides*, 27 *M. mycoides subsp. capri*, 7 *M. capricolum subsp. capripneumoniae*, 4 *M. capricolum subsp. capricolum*, 3 *M. leachii*, 1 *M. feriruminatoris*, 9 *M. hyorhinis*, 11 *M. hyopneumoniae*, 24 *M. gallisepticum*, 2 *M. pulmonis*, 89 *M. pneumoniae*, 6 *M. genitalium*, 2 *M. cynos*, and 8 *M. canis* genomes. All accession numbers are listed in the Supplementary Table S1. We observed that one of the 8 *M. canis* genomes and six of the 55 genomes belonging to members of the “*Mycoplasma mycoides* cluster” had no significant alignment to GlpO. This was perhaps because of inter-strain variation or due to poor quality draft genomes lacking the GlpO region. Given the cut-off we used in our analysis, we first observed that all GlpO sequences were conserved, even considering *Mycoplasma* species spanning the whole class of *Mollicutes* (Figure 1). When present in a given species, the GlpO sequences were highly conserved (Figure 1). This statement holds true for all mycoplasma species tested, including all members of the “*Mycoplasma mycoides* cluster.” We then assessed the presence of the genes involved in glycerol metabolism, namely the *glpFKO* and the *gtsABCD* clusters across the *Mollicutes* class, which mainly includes obligate parasites found in a wide spectrum of hosts ranging from plants, insects, animals, and humans. The results of this comparative genomic analysis are shown in Figure 2. Our analyses revealed that the structures for both clusters are not perfectly conserved with one of the genes being physically separated from the rest in several species including *M. hyopneumoniae* strain 232, *M. pulmonis* strain UAB CTIP and *M. genitalium* G37. Furthermore, the genes included in the two clusters were not consistently present across the entire *Mollicutes* class, with at least one gene missing in several species. For instance, the entire *glpFKO* cluster is completely missing in several species of the cluster Hominis (i.e., *M. hominis* and *M. synoviae*) (H, Figure 2), several *Ureaplasma* strains and in the Acholeplasma/Phytoplasma cluster (AAP, Figure 2). However, the clusters have been retained throughout the Spiroplasma group (S, Figure 2) except for *Mesoplasma florum* L1 and *Spiroplasma citri* GII-3, which are completely lacking the *glpFKO* cluster, at least in the strains analyzed here. The same observations were found for the *gtsABCD* cluster in which *gtsA* appear to be missing in several species of the Hominis and Pneumoniae phylogenetic clusters. Additionally, the entire *gtsABCD* cluster was found conserved in species where the *glpO* gene was absent, including *M. bovis* strain PG45 and *M. agalactiae* strain PG2 (Figure 2). Strikingly, both clusters are fully retained in all of the examined members of the “*Mycoplasma mycoides* cluster” (Mc, Figure 2) allowing for the cellular localization of the GlpO enzyme to be comprehensively assessed across the entire cluster.

### Generation of the Mutant GM12::YCpMmyc1.1-*ΔglpO* and Verification of the Specificity of the Rabbit Anti-GlpO Polyclonal Antibody

**FIGURE 3:**
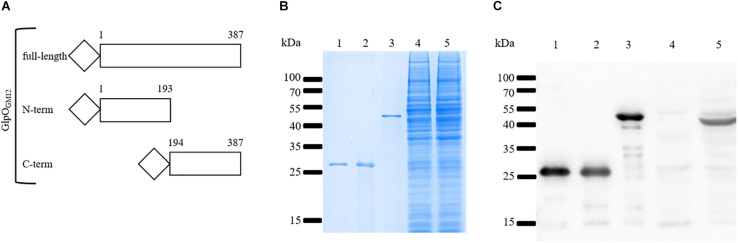
Specificity of the anti-GlpO serum. **(A)** Schematic representation of the three histidine-tagged GlpO peptides produced in *E. coli*. **(B)** Purified GlpO_MmcGM12_ N-term (lane 1), GlpO_MmcGM12_ C-term (lane 2), GlpO_MmcGM12_ full-length (lane 3) as well as total proteins of GM12::YCpMmyc1.1-*ΔglpO* (lane 4) and GM12::YCpMmyc1.1 (lane 5) were checked by SDS-PAGE and visualized using Coomassie staining. **(C)** Immunoblot was performed on the same samples previously described (lanes 1–5) using the anti-GlpO serum.

To properly investigate the cellular location of GlpO in the “*Mycoplasma mycoides* cluster,” we decided to produce an isogenic mutant strain, namely GM12::YCpMmyc1.1-*ΔglpO*, derived from GM12::YCpMmyc1.1 using the TREC technique (see section “Materials and Methods”). In addition to verifications at the genomic level including PFGE and whole genome sequencing, we performed further characterizations of our mutant strain by confirming the absence of GlpO-specific bands via immunoblotting using an anti-GlpO antibody (Figure 3C, lane 4).

The anti-GlpO antibody was originally produced by immunization of rabbits with the GlpO protein from the *M. mycoides* subsp. *mycoides* (*Mmm*) Afadé strain (Pilo et al., 2005). We anticipated that this GlpO polyclonal antibody would be able to detect GlpO from all “*Mycoplasma mycoides* cluster” species due to their high degree of GlpO aa sequence similarity (Liljander et al., 2019). Nevertheless, to verify that the anti-GlpO antibody shows high specificity for the detection of the GlpO protein, we produced the following recombinant *Mmc* proteins in *E. coli*: GlpO_GM12_ (1-387 aa), NT-GlpO_GM12_ (1-193aa), and CT-GlpO_GM12_ (194-387aa) as depicted in Figure 3A. Since GlpO is reportedly a surface-located protein, we opted to produce these three recombinant GlpO to avoid any potential problems during protein purification in *E. coli*. In fact, there were no issues in the expression and purification of these three constructs since all were highly soluble. All three recombinant proteins (Figures 3B,C, lanes 1–3), along with cell lysates of our GM12::YCpMmyc-*ΔglpO* mutant strain and its parental strain (Figures 3B,C, lanes 4 and 5), were separated by SDS-PAGE followed by Coomassie brilliant blue gel staining (Figure 3B) or immunoblotting using the anti-GlpO antibody (Figure 3C). Figure 3B shows that the recombinant proteins have high purity and display their anticipated molecular weight, while the proteins belonging to the cellular lysates of the *Mmc* strains appear to be equally loaded. Figure 3C shows that the GlpO-specific signals are at the expected sizes for the three recombinant GlpO proteins (lanes 1–3), which was also confirmed by immunoblotting with anti-his antibody (data not shown). More importantly, a GlpO-specific signal at 42 kDa was observed from total protein of the parental strain GM12::YCpMmyc1.1 (Figure 3C, lane 5), while no signal corresponding to GlpO was obtained in the total protein of GM12::YCpMmyc1.1-*ΔglpO* (Figure 3C, lane 4). This experiment confirmed the absence of GlpO gene since no corresponding GlpO protein was detected in the GM12::YCpMmyc1.1-*ΔglpO* lysate and it verified the ability of the anti-GlpO antibody to recognize GlpO. However, several additional and likely unspecific faint bands were also detected in the cell lysate of *Mmc*.

### The GM12::YCpMmyc1.1-*ΔglpO* Is Not Able to Produce H_2_O_2_ *in vitro* in Comparison to Other Mycoplasma Species, Including *M. feriruminatoris*

**FIGURE 4:**
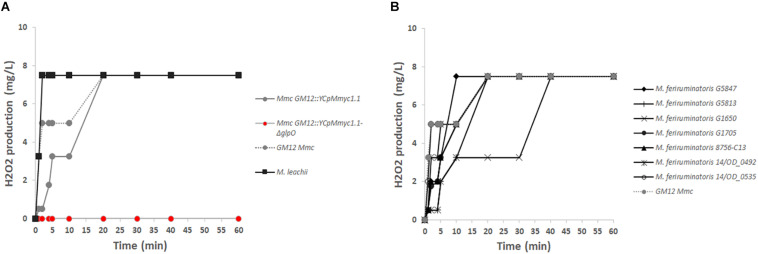
*In vitro* production of hydrogen peroxide. **(A)** The inability of the GM12::YCpMmyc1.1-*ΔglpO* mutant strain to produce H_2_O_2_ was compared to its parental strain GM12::YCpMmyc1.1 as well as to the wt *Mmc* GM12 and *M. leachii* PG50. **(B)** The capacity of *Mycoplasma feriruminatoris* to produce H_2_O_2_ was assessed using the type strain G5847^T^ as well as six additional field strains detailed in Table 1.

To further validate the true nature of the GM12::YCpMmyc1.1-*ΔglpO* mutant strain, we also tested its inability to produce H_2_O_2_ *in vitro*, in the presence of glycerol. The GM12::YCpMmyc1.1-*ΔglpO* mutant produced no detectable amount of H_2_O_2_ in the presence of glycerol compared to its parental strain, even up to 1 h after the addition of glycerol (Figure 4A). Specifically, we observed that H_2_O_2_ production of GM12::YCpMmyc1.1 was comparable to that of the wild-type strain GM12. A H_2_O_2_ concentration above 2 mg/L was quantified 5 min after the addition of glycerol in both cases and plateaued at 8 mg/L after 20 min of incubation. This high concentration of H_2_O_2_ was already reached 2 min post-glycerol addition in the cells of *Mycoplasma leachii* (Figure 4A). These results were further verified using a qualitative colorimetric on-the-plate H_2_O_2_ assay (Supplementary Materials and Methods and Supplementary Figure S1) using HRP and 3,3′-diaminobenzidine tetrahydrochloride hydrate (DAB). H_2_O_2_ production is revealed by the colonies adopting a red-brown color due to the oxidation of DAB. Colonies of *M. leachii*, *Mmc* GM12 strain and GM12::YCpMmyc1.1 were all stained dark brown within 10 min (Supplementary Figures S1A–C, respectively) while the GM12::YCpMmyc1.1-*ΔglpO* mutant colonies remained white (Supplementary Figure S1D). Since *M. feriruminatoris* has all of the necessary genes required for H_2_O_2_ production (Figure 2), we examined H_2_O_2_ levels from seven *M. feriruminatoris* strains, including the previously characterized strain G5847^T^ (Table 1), which was previously reported to not produce H_2_O_2_ (Jores et al., 2013). Interestingly, we found that all seven strains produced detectable amounts of H_2_O_2_, albeit with varying kinetics (Figure 4B). Collectively, these results genetically and phenotypically confirmed the proper deletion of *glpO* in our GM12::YCpMmyc1.1-*ΔglpO* mutant strain.

### Scanning Electron Microscopy Experiments Could Not Pinpoint the Localization of GlpO_GM12_

**FIGURE 5:**
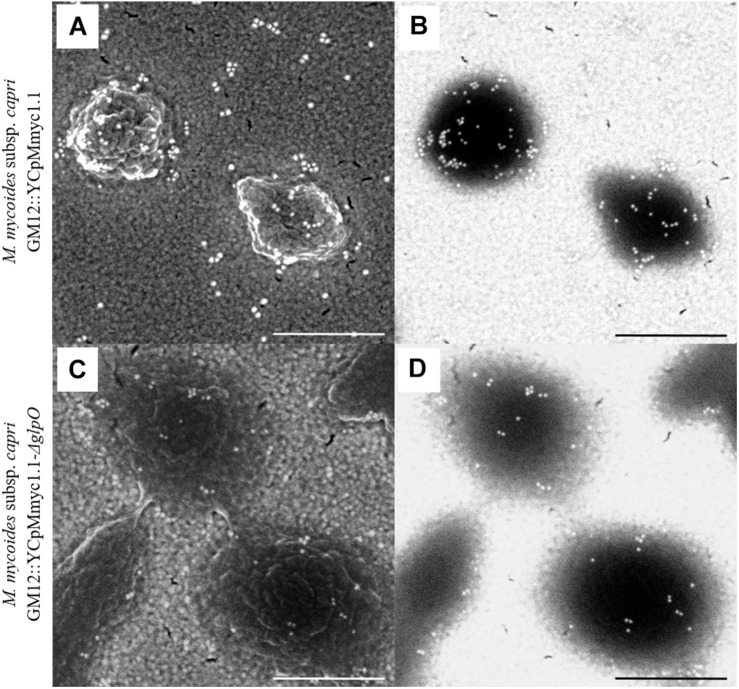
Scanning electron micrographs showing immunogold labeling of the parental strain GM12::YCpMmyc1.1 **(A,B)** as well as the mutant strain GM12::YCpMmyc1.1-*ΔglpO* **(C,D)** incubated with anti-GlpO serum. Secondary electron micrographs show the cell surface of the mycoplasmas **(A,C)**. Back-scattered electron micrographs reveal the presence of the gold-conjugated secondary antibody **(B,D)**. Scale bar, 500 nm.

In order to assess the cellular localization of GlpO, we performed SEM on GM12::YCpMmyc1.1-*ΔglpO* and its parental strain GM12::YCpMmyc1.1 as previously described (Pilo et al., 2005) (Figure 5). Secondary electron micrographs revealed intact *Mycoplasma* cells without any particular phenotype associated with the GlpO deletion (Figures 5A,C). Labeling of the mycoplasma cell surface was observed when cells from both strains were incubated with anti-GlpO polyclonal antibody followed by an anti-rabbit secondary antibody conjugated with gold particles (Figures 5B,D). However, we observed a similar labeling pattern in both strains, regardless of the presence or absence of GlpO, indicating that this labeling is not GlpO-specific and is probably due to unspecific recognition of other surface-located mycoplasma proteins by the polyclonal antibody. The specificity of the secondary antibody was corroborated by negative controls, in which the primary antibody was omitted, or an irrelevant primary antibody was used instead. These controls showed no labeling of the cells (data not shown). As labeling occurred irrespective of the strain, the SEM results obtained here using this specific anti-GlpO antibody are not meaningful with respect to the localization of GlpO on whole *Mycoplasma* cells.

### *In silico* Analyses Strongly Support the Cytoplasmic Localization of GlpO

We performed various *in silico* analyses using GlpO sequences from different strains of the “*Mycoplasma mycoides* cluster” in order to gain information concerning GlpO localization. To this end, we first searched for putative transmembrane (TM) domains or signal peptides on the GlpO sequences using TMHMM2.0 and SignalP 5.0. At first, we did not find any TM domains in any of the GlpO aa sequences tested, including the GlpO sequence derived from the *Mmm* Afadé strain (Supplementary Figure S2A). However, one N-terminal TM domain was predicted for the already characterized membrane lipoprotein, LppQ (Supplementary Figure S2B) and six TM regions for the membrane-associated glycerol facilitator transporter, GlpF (Supplementary Figure S2C), thus demonstrating the capacity of the software to detect such domains in mycoplasma protein sequences. Interestingly, a putative TM domain was detected using TMHMM2.0 on the N-terminal part of several GlpO sequences of *Mycoplasma gallisepticum* including the one from the strain R_high_ (Supplementary Figure S2D), *Mycoplasma canis* PG14 (Supplementary Figure S2E) and *Mycoplasma cynos* C142 (Supplementary Figure S2F). However, none of these putative domains was confirmed as signal peptides whereas the signal peptide previously identified in LppQ amino acids sequence was identified with high confidence (data not shown). We also used different prediction software (i.e., Phobius, Psi-Pred, LipoP) and all gave similar and consistent predictions. The prediction software TMpred, which was previously used to predict 2 TM regions in the GlpO sequence of *Mmm* Afadé (Pilo et al., 2005), is the only software tested that indicated the presence of putative TM domains in GlpO sequences of the “*Mycoplasma mycoides* cluster” even if the scores attributed to each of them were rather low (Supplementary Figure S3). In order to provide a more comprehensive overview of GlpO subcellular localization across the “*Mycoplasma mycoides* cluster” and other closely related species, we analyzed all GlpO sequences previously used (Figure 1 and Supplementary Table S1) using PSORTb v3.0, as this software allows fine tuning for *Mycoplasma* species. All GlpO sequences were predicted to be cytoplasmic proteins except for the GlpO sequences belonging to *M. gallisepticum and M. hyopneumoniae*, for which the predictions were inconclusive (Table S1). Collectively, these *in silico* analyses argue in favor of a cytoplasmic localization for GlpO, at least in the “*Mycoplasma mycoides* cluster”.

### Triton X-114 Fractionations Reveal That GlpO Is a Cytoplasmic Protein in All Species of the “*Mycoplasma mycoides* Cluster”

**FIGURE 6:**
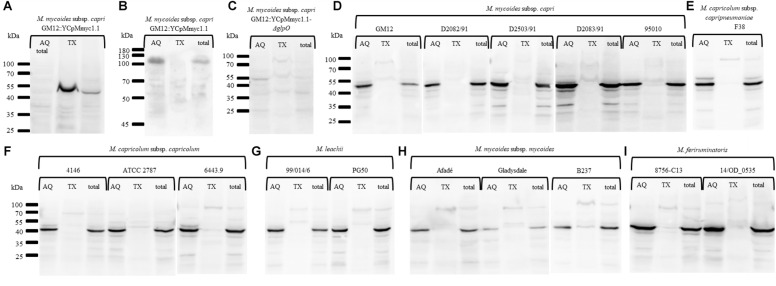
Immunoblots performed after Triton X-114 phase partitioning showing the *in vitro* cytoplasmic localization of GlpO in the “*Mycoplasma mycoides* cluster.” Immunoblots were carried out using an anti-LppQ antibody **(A)**, the anti-beta Galactosidase polyclonal serum **(B)** or the anti-glpO antibody **(C–H)** on total proteins of GM12::YCpMmyc1.1 **(A,B)**, GM12::YCpMmyc1.1-*ΔglpO* **(C)**, five different strains of *M. mycoides* subsp. *capri* **(D)**, two strains of *M. feriruminatoris* **(E)**, three strains of *M. capricolum* subsp. *capricolum* **(F)**, two strains of *M. leachii* **(G)**, three strains of *M. mycoides* subsp. *mycoides* **(H)**, and one strain of *M. capricolum* subsp. *capripneumoniae* **(I)**.

To specifically investigate whether GlpO is membrane-associated or resides in the cytoplasm, we performed Triton X-114 fractionations with representative species across the “*Mycoplasma mycoides* cluster” (Table 1 and Figure 6). First, phase separation of integral membrane proteins of the GM12::YCpMmyc1.1 parental strain followed by probing with an anti-LppQ antibody resulted in a LppQ-specific signal at ∼53 kDa exclusively in the detergent fraction (TX) as expected (Figure 6A). The same partitioning was also followed by probing with an anti-beta Galactosidase polyclonal serum. This protein, present of the GM12::YCpMmyc1.1 genome, is a known cytoplasmic protein. As expected, we obtained a signal at ∼120 kDa specific for the *E. coli* beta-Galactosidase only in the aqueous (AQ) soluble fraction (Figure 6B). Altogether, these results demonstrated the pertinence of our Triton X-114 phase partitioning experiments. No GlpO-specific signal was obtained when proteins of each fraction of the GM12::YCpMmyc1.1-*ΔglpO* mutant strain were separated and probed using anti-GlpO (Figure 6C). The same TX-114 phase partitioning experiments were then conducted on different species of the “*Mycoplasma mycoides* cluster” including five *Mmc* strains (Figure 6D), one *Mycoplasma capricolum* subsp. *capripneumoniae* strain (Figure 6E), three *Mycoplasma capricolum* subsp. *capricolum* strains (Figure 6F), two *Mycoplasma leachii* strains (Figure 6G), three *Mycoplasma mycoides* subsp. *mycoides* strains (Figure 6H) and two *Mycoplasma feriruminatoris* strains (Figure 6I). All immunoblots revealed a consistent GlpO-specific signal at ∼42 kDa exclusively in all the AQ fractions, as well as in the total fractions. No clear GlpO-specific signal was observed in any of the insoluble fractions. Additionally, tryptic shaving experiments were conducted on three strains previously included in the Triton X-114 experiments, namely *M. feriruminatoris* G5847^T^, *Mmc* GM12 and GM12::YCpMmyc1.1. We did not find any reduction in GlpO-specific signals when any of the aforementioned strains were incubated with trypsin (Supplementary Figure S4). Thus, our *in vitro* experiments clearly showed that GlpO is a cytoplasmic enzyme in all tested species of the “*Mycoplasma mycoides* cluster.”

## Discussion

In this study, we re-evaluated the cellular localization of GlpO across the whole “*Mycoplasma mycoides* cluster” using a combination of *in silico* and *in vitro* approaches. GlpO was first described as a surface-exposed virulence factor in *Mmm* Afadé (Pilo et al., 2005). While synthetic genomics tools can be applied to several mycoplasma species, *Mmm* is still refractory to genome transplantation impairing the use of such tools for its study (Labroussaa et al., 2016). Therefore, the construction of an isogenic mutant GM12::YCpMmyc1.1-*ΔglpO* was carried out in the phylogenetically closely related subspecies *Mmc*. The GM12::YCpMmyc1.1-*ΔglpO* mutant and its parental strain were used to assess GlpO localization using SEM analysis. The surface labeling observed for the mutant strain, despite the absence of the GlpO protein, was the same as the one observed for its parental strain (Figure 5). This clearly indicates unspecific binding of the anti-GlpO serum to other surface-exposed *Mmc* proteins. This is consistent with the fact that unspecific bands were observed on all immunoblots carried out using proteins of the GM12::YCpMmyc1.1-*ΔglpO* mutant and its parental strain (Figures 3, 4). The specificity of the anti-GlpO serum could not be assessed in any of the SEM negative controls included in the original study (Pilo et al., 2005), and also repeated in this study. However, the use of the GM12::YCpMmyc1.1-*ΔglpO* isogenic mutant greatly facilitated the interpretation of the results. In addition to whole genome sequencing, H_2_O_2_ quantification confirmed the deletion of the *glpO* gene as this mutant was unable to produce H_2_O_2_ as previously reported (Tsarmpopoulos et al., 2016). Interestingly, all *M. feriruminatoris* strains tested including the type strain G5847^T^ as well as six field strains, were able to produce detectable amounts of H_2_O_2_, in contrast to previous findings using the type strain G5847^T^ (Jores et al., 2013).

Total protein content of 16 mycoplasma strains belonging to six different species, and including three *Mmm* strains were separated using Triton X-114 phase partitioning followed by anti-GlpO immunoblotting. GlpO was only detected in the soluble fraction after phase separation confirming its cytoplasmic localization. GlpO was reported as a predominantly cytoplasmic enzyme in *M. pneumoniae* but a dual localization was suggested due to a faint GlpO-specific signal observed in the insoluble fraction (Hames et al., 2009). In our study, a GlpO-specific signal was only observed in the insoluble fraction when the sample temperature was not kept in accordance with the protocol prior to the fractionation. GlpO was previously shown to be a cytoplasmic protein in other bacteria including *Enterococcus casseliflavus* (Claiborne, 1986) and many lactic-acid bacteria which also use glycerol as a carbon or energy source (Baureder and Hederstedt, 2013).

The reallocation of GlpO to the cytoplasm for the members of the “*Mycoplasma mycoides* cluster” implies that mycoplasmas need to export the generated H_2_O_2_ to minimize self-toxicity. In the absence of H_2_O_2_-degrading enzymes in mycoplasmas, such as catalase or superoxide dismutase, H_2_O_2_ could potentially diffuse freely outside of the mycoplasma cells even if its diffusion is known to be limited (Bienert et al., 2007). A peroxiredoxin, belonging to a family of antioxidant enzymes (EC 1.11.1.15) able to reduce H_2_O_2_, was identified in *Mycoplasma hyopneumoniae* (Machado et al., 2009). Orthologs have been identified in other mycoplasmas, including species of the “*Mycoplasma mycoides* cluster” such as *Mmc* strain GM12 (MMCAP2_0054), *Mmc* strain 95010 (MLC_0500), *Mmm* strain PG1 (MSC_0053), but no precise function in cell detoxification has been proposed in these organisms. Alternatively, transporters such as GlpF, the GtsABC proteins or a yet to be discovered one, could be used to actively pump H_2_O_2_ outside of the cells. It is reasonable to assume that GlpO is likely to be located in close vicinity of this putative transporter in order to facilitate the export of H_2_O_2_. Therefore, if H_2_O_2_ is injected in the host cells as previously suggested for *Mmm* (Pilo et al., 2005), then perhaps H_2_O_2_ production is only activated only when mycoplasma cells are directly interacting with the host cells in order to avoid any self-generated H_2_O_2_ toxicity. Recently, GlpQ was identified as a trigger enzyme regulating the level of expression of enzymes involved in different physiological pathways in *M. pneumoniae* (Schmidl et al., 2011). It is possible that such an enzyme might be able to trigger the H_2_O_2_ production upon interaction with host cells in other mycoplasma species. The role of increasing concentrations of H_2_O_2_ in the close vicinity of mycoplasma cells is still very speculative. Low levels of hydrogen peroxide have been shown to function as signaling molecules inducing epithelial cell death (Waghray et al., 2005) or *in vitro* NETs formation (Fuchs et al., 2007) and may also be used by mycoplasmas to escape the immune system or retrieve nutrients from its host.

It was previously assumed that virulent mycoplasma strains, producing higher amounts of H_2_O_2_, expressed GlpO at their surface to limit its toxic effect (Vilei et al., 2000; Hames et al., 2009). Even if we cannot exclude the possibility that GlpO might be translocated to the mycoplasma cell surface *in vivo*, this possibility is certainly not supported by our *in silico* and *in vitro* results. Indeed, no TM regions were detected *in silico* in any of the GlpO aa sequences belonging to members of the “*Mycoplasma mycoides* cluster” using TMHMM, which is considered to be the best performing TM prediction program (Moller et al., 2001). The only GlpO aa sequences suggested to have a TM region, even if none were confirmed as signal peptides, belong to three different mycoplasma species including *M. gallisepticum*. Despite showing cytotoxicity *in vitro*, the production of H_2_O_2_ was reported to be dispensable for the virulence of *M. gallisepticum* in the tracheas of its natural host (Szczepanek et al., 2014). This brings into question the role of H_2_O_2_ in the pathogenesis of other *Mycoplasma* species. Moreover, the presence of both the *glpFKO* and *gtsABCD* genes were suggested to facilitate a higher level of glycerol uptake ultimately leading to a higher production of H_2_O_2_ (Vilei and Frey, 2001). However, our *in silico* data do not support such a correlation since it is frequent to find pathogenic mycoplasma species in which at least one of these two pathways is absent, if not both (Figure 2). As previously shown in several species of spiroplasmas, independent events leading to the loss of the genes involved in these clusters are likely to explain this phenomenon (Chang et al., 2014). To the best of our knowledge, the role of H_2_O_2_ as a virulence factor has never been confirmed *in vivo* for any of the species belonging to the “*Mycoplasma mycoides* cluster.” A challenge experiment using a recently described infection model for *Mmc* GM12 in goats [31], employing the mutant GM12::YCpMmyc1.1-*ΔglpO* and its parental strain would help to clarify whether GlpO is a true virulence trait in *Mycoplasma mycoides*.

Overall, our results certainly modify the current concept of H_2_O_2_ production in the “*Mycoplasma mycoides* cluster.” Due to its novel cytoplasmic localization, as determined by our *in vitro* studies, it is very unlikely that GlpO can continue to be considered as a candidate antigen to induce a protective response in the host in the future. A previous attempt to raise such a protection in cattle using a recombinant GlpO from *Mmm* failed to do so (Mulongo et al., 2013) and it is likely to be the case in other species. However, hosts’ antibodies from convalescent animals that survived an *Mmc* infection have been shown to reduce H_2_O_2_ production *in vitro* raising the question about the role of other proteins involved in the glycerol uptake (Liljander et al., 2019). The generation of a series of isogenic mutants for all the proteins involved in this complex pathway should shed light on the exact role of this pathway in mycoplasmas.

## Data Availability

The datasets generated for this study can be found in Genbank, GCA_900489625.

## Author Contributions

JJ and FL designed the study. MS, PN, JJ, and FL drafted the manuscript. SC, LM, and SV constructed the GM12::YCpMmyc1.1-*ΔglpO* mutant strain. MS performed the Triton X-114 phase partitioning and corresponding immunoblots. PN purified the proteins from *E. coli* and performed the *in vitro* and on-the-plate H_2_O_2_ assays as well as tryptic shaving experiments. MS and MHS carried out the SEM analyses. FL and AD performed the *in silico* analyses. All authors read and approved the final manuscript.

## Conflict of Interest Statement

The authors declare that the research was conducted in the absence of any commercial or financial relationships that could be construed as a potential conflict of interest.

## Footnotes

1
https://services.cbib.u-bordeaux.fr/molligen/

2
http://mbgd.genome.ad.jp/

3
http://www.cbs.dtu.dk/services/TMHMM/

4
https://embnet.vital-it.ch/software/TMPRED_form.html

5
http://www.cbs.dtu.dk/services/SignalP/

6
https://www.psort.org/psortb/

## Supplementary Material

TABLE S1 GlpO sequences used in this study. Each GlpO amino acids sequence is provided with information regarding its respective locus tag, mycoplasma strain, contig coordinates on the genome, protein length (amino acids), whole amino acids sequence and current annotation (columns A to F, respectively). In addition, results from the PSORTb analysis obtained for each the GlpO sequences are also included. Scores associated with the different putative localizations are indicated (columns G to J) as well as the final prediction given by the software (column K). All the GlpO sequences used to generate the unrooted phylogenetic tree are highlighted in yellow.
