# Corrigendum: Evidence for the cytoplasmic localization of the L-α-Glycerophosphate oxidase in members of the “*Mycoplasma mycoides* cluster”

**DOI:** 10.3389/fmicb.2023.1293129

**Published:** 2023-09-27

**Authors:** Melanie Schumacher, Pamela Nicholson, Michael H. Stoffel, Suchismita Chandran, Adonis D'Mello, Li Ma, Sanjay Vashee, Joerg Jores, Fabien Labroussaa

**Affiliations:** ^1^Institute of Veterinary Bacteriology, University of Bern, Bern, Switzerland; ^2^Division of Veterinary Anatomy, University of Bern, Bern, Switzerland; ^3^J. Craig Venter Institute, Rockville, MD, United States; ^4^Institute for Genome Sciences, University of Maryland School of Medicine, Baltimore, MD, United States

**Keywords:** “*Mycoplasma mycoides* cluster”, synthetic genomics, mycoplasma virulence traits, L-α-glycerophosphate oxidase, Triton X-114, scanning electron microscopy

In the published article, there were several errors in Table 1 as published. The host species from several *Mycoplasma feriruminatoris* was wrongly indicated as “Goat”. *M. feriruminatoris* strain G5813/1+2 was isolated from a Rocky Mountain Goat, whereas the six other *M. feriruminatoris* strains were isolated from Alpine Ibex. In addition, the *Mycoplasma mycoides* subsp. *capri* strain D2082/91 is also referred as strain D2482/91 elsewhere and both names are now indicated. The year of isolation of the two *M. feriruminatoris* strains 14/OD_0492 and 14/OD_0535 was wrongly indicated as “1994” and is now updated to “2014”. Finally, several references appearing in Table 1 were wrongly stated as “This study”. The reference for the two *Mycoplasma mycoides* subsp. *capri* strain D2503/91 and D2082/91 is now indicated as Vilei et al. (2006). The reference for the two *Mycoplasma feriruminatoris* strain G5813/1+2 and G1705 is now indicated as Fischer et al. (2012). The references for the *Mycoplasma feriruminatoris* strains 8756-C13 and G1650 are now indicated as Manso-Silván et al. (2007) and Schnee et al. (2011), respectively.

The corrected Table 1 and its caption appear below.

**Table 1:** *Mycoplasma* species used in this study.

***Mycoplasma* species**	**Strain**	**Origin**	**Isolated**	**Host**	**Accession number**	**Reference**
*M. leachii*	99/014/06	Australia	1999	Calf	FR668087.1	Djordjevic et al., 2001
*M. leachii*	PG50^T^	Australia	1963	Cattle	CP002108.1	Wise et al., 2012
*M. mycoides* subsp. *capri*	D2083/91	Switzerland	1991	Goat		Thiaucourt et al., 2000
*M. mycoides* subsp. *capri*	95010	France	1995	Goat	FQ377874.1	Thiaucourt et al., 2011
*M. mycoides* subsp. *capri*	D2503/91	Switzerland	1991	Goat		Vilei et al., 2006
*M. mycoides* subsp. *capri*	D2082/91; D2482/91	Switzerland	1991	Goat		Vilei et al., 2006
*M. mycoides* subsp. *capri*	GM12	USA	1979	Goat	CP001621.1	Lartigue et al., 2009
*M. capricolum* subsp. *capricolum*	6443.90	France	1990	Goat		Vilei et al., 2006
*M. capricolum* subsp. *capricolum*	4146	France	1980	Goat		Fischer et al., 2012
*M. capricolum* subsp. *capricolum*	California kid^T^; ATCC 27343	USA	1955	Goat	CP000123.1	Cordy et al., 1955
*M. feriruminatoris*	G5847^T^	Berlin	1993	Alpine Ibex		Jores et al., 2013
*M. feriruminatoris*	8756-C13	USA	<1987	Rocky Mountain Goat		Manso-Silván et al., 2007
*M. feriruminatoris*	G5813/1+2; 322/93	Berlin	1993	Alpine Ibex		Fischer et al., 2012
*M. feriruminatoris*	G1650; 27/94	Berlin	1993	Alpine Ibex		Schnee et al., 2011
*M. feriruminatoris*	G1705; 28/94	Berlin	1993	Alpine Ibex		Fischer et al., 2012
*M. feriruminatoris*	14/OD_0492	Switzerland	2014	Alpine Ibex		This study
*M. feriruminatoris*	14/OD_0535	Switzerland	2014	Alpine Ibex		This study
*M. capricolum* subsp. *capripneumoniae*	F38^T^	Kenya	1976	Goat	LN515398.1	Falquet et al., 2014
*M. mycoides* subsp. *mycoides*	Afadé	Cameroon	1968	Cattle	LAEX01	Fischer et al., 2015
*M. mycoides* subsp. *mycoides*	Gladysdale	Australia	<1964	Cattle	CP002107	Wise et al., 2012
*M. mycoides* subsp. *mycoides*	B237	Kenya	1987	Cattle	LAEW01	Fischer et al., 2015

The authors apologize for these errors and state that these do not change the scientific conclusions of the article in any way. The original article has been updated.
