# Supplementary material for: Evidence for the Cytoplasmic Localization of the L-α-Glycerophosphate Oxidase in Members of the “Mycoplasma mycoides Cluster”
Source: Front Microbiol. 2019 Jun 19;10:1344. doi: 10.3389/fmicb.2019.01344 (PMC6593217; doi:10.3389/fmicb.2019.01344)
Supplement: Supplementary file 3 [file Table_1.DOCX]

**L-α-glycerophosphate oxidase (GlpO) is a cytoplasmic enzyme in members of the ‘*Mycoplasma mycoides* cluster’**

Melanie Schumacher^1#^, Pamela Nicholson^1#^, Michael H. Stoffel^2^, Suchismita Chandran^3^, Li Ma^3^, Sanjay Vashee^3^, Joerg Jores^1*^ and Fabien Labroussaa^1*§^

^1^Institute of Veterinary Bacteriology, University of Bern, PO Box, CH-3001 Bern, Switzerland

^2^Division of Veterinary Anatomy, University of Bern, PO Box, CH-3001 Bern, Switzerland

^3^J. Craig Venter Institute, 9605 Medical Center Drive, MD 20850 Rockville, USA

# contributed equally as first authors

^*^contributed equally as last authors

^§^corresponding author.

**Supplementary Data**

Supplementary Materials and Methods page 2-3

Supplementary Figure S1 page 4

Supplementary Figure S2 page 5

Supplementary Figure S3 page 6

Supplementary Figure S4 page 7

Supplementary References page 8

**Supplementary Materials and Methods**

**On-the-plate H_2_O_2_ test**

A qualitative on-the-plate screening of H_2_O_2_ production was developed based on a previously reported assay (Rice et al., 2001). The designated *Mycoplasma* strains were grown on agar medium plates (60 mm diameter) and grown at 37 °C until clearly visible colonies were obtained. Subsequently, 400 μL freshly prepared and filtered Peroxide Detection Solution (PDS) (calcium chloride, 0.06 g/L; potassium chloride, 0.05 g/L; sodium bicarbonate, 0.025 g/L; sodium chloride, 2.25 g/L; 3,3'-diaminobenzidine (DAB, Sigma), 6 mg/mL; horseradish peroxidase (HRP, Sigma), 1 mg/mL; glycerol 0.05 g/mL) was spread carefully onto the surface of the plates and incubated for 10 minutes. *Mycoplasma* colonies producing H_2_O_2_ adopt a red-brown colour. The assay was developed and validated by performing the assay in the absence of HRP, DAB, or glycerol as well as performing the assay with various well-known H_2_O_2_ producing *Mycoplasma* spp. such as *M. leachii*.

**Tryptic shaving experiments**

Experiments were performed as previously described (Nicholson et al., 2019). Four mL of each designated *Mycoplasma* culture were split equally and the bacterial pellets were either re-suspended in 2 mL of incubation buffer (20 mMTris-HCl, 150 mM NaCl, 10mM CaCl_2_ and 0.5 M sucrose) supplemented with 10 µg trypsin reconstituted in 100 µL acetic acid (Promega V5111) or in 2 mL of incubation buffer supplemented with 100 µL acetic acid but lacking trypsin. All samples were then incubated at 37 °C for 2 h. Thereafter the samples were centrifuged at 10'000 x g for 15 min at 4 °C. The supernatants were retained, and the cells were re-suspended in cold buffer (20 mM Tris-HCl, 150 mM NaCl, pH 7.6). The cells and supernatant samples were mixed 1:1 with 2 x sample buffer and SDS-PAGE followed by immunoblotting was performed as detailed in the materials and methods section of the main paper.

**Supplementary Results**


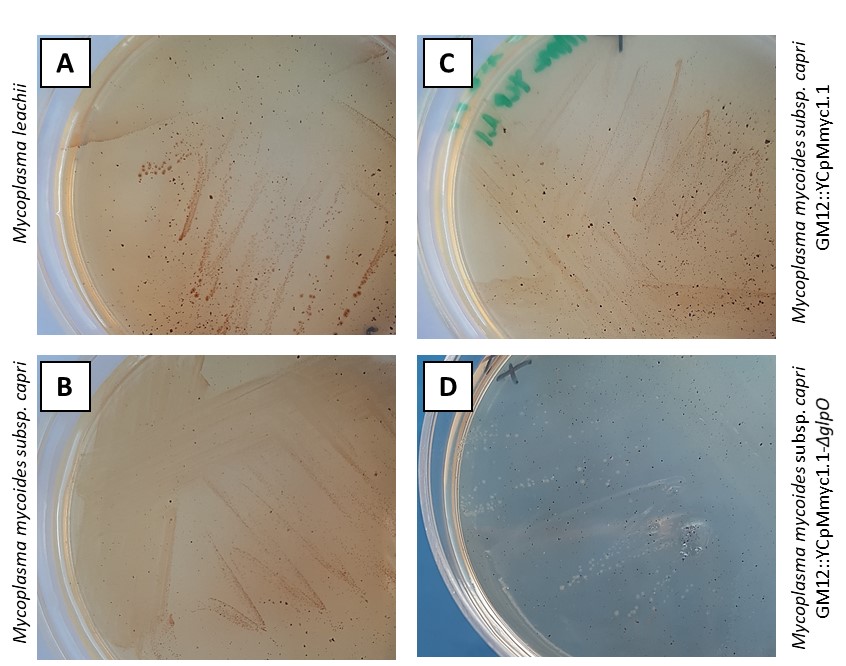


**Supplementary Figure S1.** GM12::YCpMmyc1.1-*ΔglpO*  mutant colonies do not produce H_2_O_2_. Detection of H_2_O_2_ production by colonies using a qualitative colorimetric on-the-plate assay wherein the production of H_2_O_2_ is revealed by the colonies adopting a red-brown color due to the oxidation of DAB in the presence of HRP, glycerol and H_2_O_2_. Mycoplasma agar plates containing colonies of (A) *M. leachii*, (B) *Mmc* GM12, (C) GM12::YCpMmyc1.1 and (D) GM12::YCpMmyc1.1-*ΔglpO*  were treated with freshly prepared Peroxide Detection Solution and the results were documented after 10 min.

**
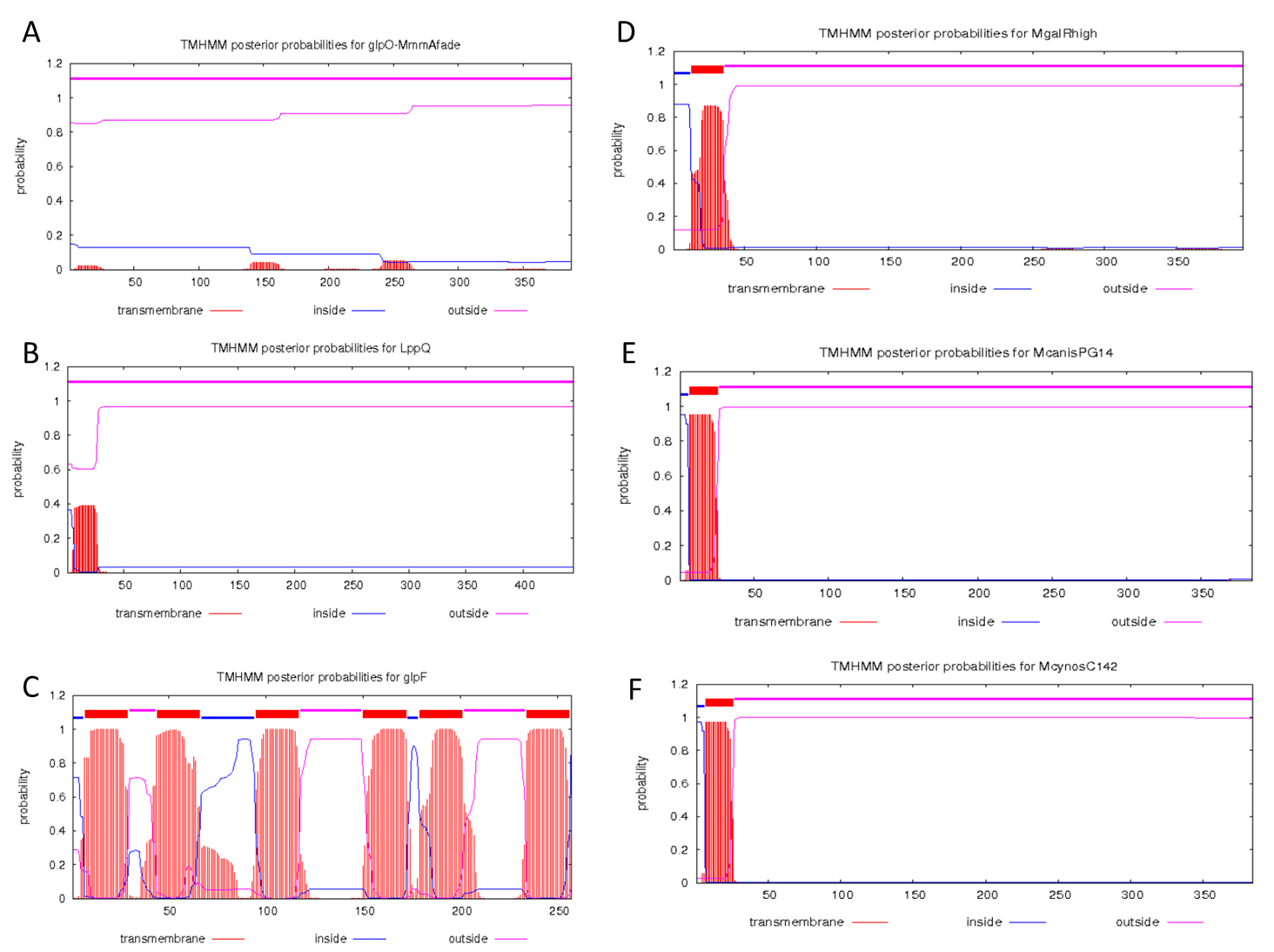
**

**Supplementary Figure S2.** *In silico* prediction of transmembrane domains (TM) using TMHMM2.0. Amino acid sequences of GlpO from *Mmm* strain Afadé (A), LppQ (B) and GlpF (C) are shown. TM predictions for GlpO sequences of *M.gallisepticum* strain Rhigh (D), *M. canis* strain PG14 (E) and *M. cynos* strain C142 (F) are also depicted.

**
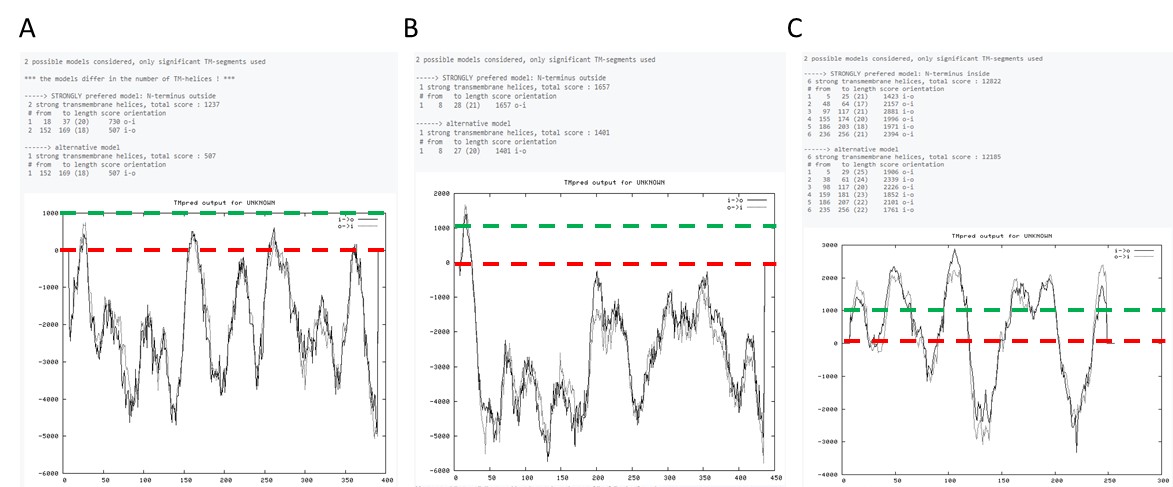
**

**Supplementary Figure S3.** *In silico* prediction of transmembrane domains (TM) using TMPred. Amino acid sequences of GlpO from *Mmm* strain Afadé (A), LppQ (B) and GlpF (C) are shown. The red line indicates a null score predicted by the software whereas the green line indicates a score of 1000.

**
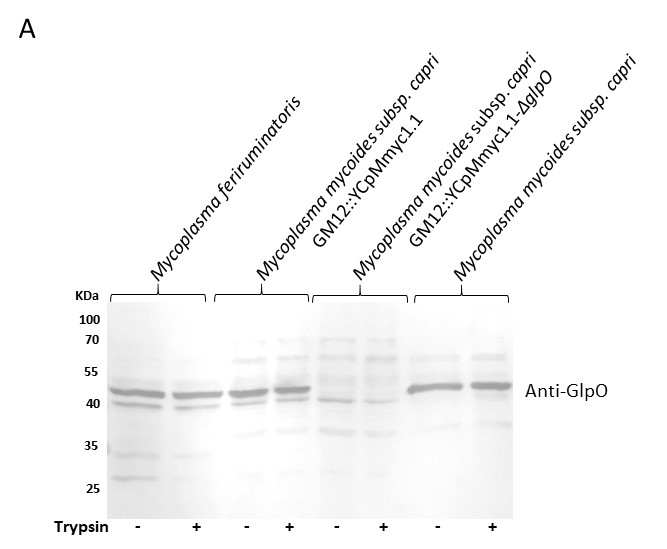
**

**Supplementary Figure S4.** Tryptic shaving of *Mycoplasma* cells revealed no difference in GlpO signal compared to unshaved cells. The designated *Mycoplasma* *mycoides* cluster strain was grown and then equally split in to two samples. The samples were incubated either with (+) or without (-) trypsin for 120 min followed by separation of the cellular proteins by SDS-PAGE followed by immunoblotting using anti-GlpO serum.

**References**

Nicholson, P., Furrer, J., Hässig, M., Strauss, C., Heller, M., Braga-Lagache, S., et al. (2019). Production of neutralizing antibodies against the secreted *Clostridium chauvoei* toxin A (CctA) upon blackleg vaccination. *Anaerobe* 56, 78–87. doi:10.1016/J.ANAEROBE.2019.02.011.

Rice, P., Houshaymi, B. M., Abu-Groun, E. A. M., Nicholas, R. A. J., and Miles, R. J. (2001). Rapid screening of H2O2 production by *Mycoplasma mycoides* and differentiation of European subsp. *mycoides* SC (small colony) isolates. *Vet. Microbiol.* 78, 343–351. doi:10.1016/S0378-1135(00)00305-9.
